# Functional Magnetic Resonance Imaging Correlations Between Fatigue and Cognitive Performance in Patients With Relapsing Remitting Multiple Sclerosis

**DOI:** 10.3389/fpsyt.2019.00754

**Published:** 2019-10-29

**Authors:** Dessislava Iancheva, Anastasya Trenova, Stefka Mantarova, Kiril Terziyski

**Affiliations:** ^1^Department of Neurology, Medical University Plovdiv, Plovdiv, Bulgaria; ^2^Military Medical Academy-MHAT Plovdiv, Sofia, Bulgaria; ^3^Department of Pathophysiology, Medical University Plovdiv, Plovdiv, Bulgaria

**Keywords:** fMRI, Multiple Sclerosis, Cognition, Fatigue, MFIs

## Abstract

The correlation between fatigue and cognitive performance in multiple sclerosis (MS) is well reported, but the intimate mechanisms of the fatigue impact on cognition are not fully defined yet. The aim of this study is to investigate blood oxygen level–dependent (BOLD) activations in relapsing remitting MS (RRMS) patients with and without cognitive dysfunction and the impact of fatigue on cortical activations. Forty-two patients with RRMS were enrolled in the study. Cognitive functioning was assessed by the Symbol Digit Modalities Test (SDMT) and Paced Serial Addition Test (PASAT). A cutoff point of a total score of 55 on the SDMT was used to divide the patients into two groups: cognitively impaired (CI), SDMT score equal to or below 55 points, and cognitively preserved (CP), SMDT score above 55 points. Fatigue was assessed by the Modified Fatigue Impact Scale (MFIS). Participants were assessed with the Beck Depression Inventory (BDI) prior to inclusion in order to exclude major depressive episode. Functional Magnetic Resonance Imaging (fMRI) scanning was performed on a 3T MRI. The PVSAT (Paced Visual Serial Addition Test) paradigm was applied as a cognitive task. All functional data were analyzed with SPM12 and statistical analysis with SPSS 19.0. No statistically significant differences between CI and CP patients were found (p=0.953, p=0.322) in the MFIS and BDI score. Performance on the PASAT in CI patients was 34.07±13.721, for CP patients 46.42±11.453, and the SDMT performance in the CI patient group was 42.40±9.179, in the CP group 57.83±2.552. Between-group analysis revealed increased activations in left Brodmann area (BA) 40 in CP patients with several clusters located in the left supramarginal gyrus. Regression analysis showed increased BOLD signal in left BA 40, right BA 40, and left BA 6, associated with a higher score on MFIS. Stronger BOLD signal in left BA 31 was associated with a lower score on MFIS. Significance level was set to p<0.05, FWE (family-wise error) corrected. The differences in BOLD activations suggest the presence of cortical reorganization in our CP patients. The impact of fatigue on cortical activation during a cognitive task is demonstrated by inconformity of activated areas depending on the MFIS score. Our results suggest that activation in BA 40 may represent a mechanism for diminishing fatigue impact on cognitive functioning in CP patients.

## Introduction

Multiple sclerosis (MS) is an inflammatory demyelinating and neurodegenerative disease of the central nervous system (CNS) characterized with widespread lesions in brain and spinal cord. It is prevalent in young adults and therefore with significant social burden (1). Research in the past 30 years has indicated that cognitive impairment affects between 40% and 70% of all MS patients. The MS-related cognitive dysfunction appears in various domains such as attention, information processing speed, processing efficiency, executive functioning, and working memory. These deficits affect many aspects of daily life, thus resulting in decreased quality of life (2). Fatigue is the most common symptom in MS and is reported in over 90% of patients (2, 3). Rudroff et al. in a recent review propose the following definition for fatigue: “The decrease in physical and/or mental performance that result from changes in central, psychological and/ or peripheral factors.” The authors emphasize the conditional dependencies of all included factors, such as the task that is performed, the environmental conditions in which it is performed, and the physical and mental capacity of the individual (4). The assessment of fatigue is most often conducted with self-report questionnaires. They range from single-item scales such as a visual analogue scale (VAS) to multidimensional scales incorporating several dimensions of fatigue such as physical and mental. The Fatigue Severity Scale (FSS) and the Modified Fatigue Impact Scale (MFIS) are two multidimensional scales that are predominantly applied in studies with MS patients (4). The relationship between fatigue and cognitive performance in MS is well reported (3, 5, 6). One obstacle remains the difficulty to objectively differentiate cognitive fatigue from physical, and in addition, research has revealed little or no relationship between self-reported and objective measurements of fatigue in clinical populations (7). As a result, the intimate mechanisms of the fatigue impact on cognition are not fully defined yet. The combined assessment with neuropsychological testing and functional MRI (fMRI) has revealed an opportunity for investigating complex compensatory mechanisms involved in cognitive functioning. Translational validation of cognitive tests and the correlation with fatigue and mood in patients with relapsing remitting multiple sclerosis (RRMS) might be a stepping-stone towards better understanding of this intricate interplay between some of the most common symptoms of MS. The aim of this study is to investigate blood oxygen level–dependent (BOLD) activations in RRMS patients with and without cognitive dysfunction and the impact of fatigue on cortical activations.

## Materials and Methods

### Participants

Forty-two patients diagnosed with RRMS according to McDonald’s criteria (2017) were enrolled in the study (8). The following inclusion criteria were applied to all participants: remission phase of the disease (defined as a period of improvement or stable clinical condition for at least 3 months), age between 18 and 55 years, primary education and, treatment with first-line disease modifying therapies (interferon-beta or glatiramer acetate). Exclusion criteria were: treatment with corticosteroids 3 months prior to entering the study; exacerbation phase of MS; and known history of drug or alcohol abuse, psychiatric illness, and other chronic diseases. All patients underwent a standard neurological examination and were assessed by the Expanded Disability Status Scale (EDSS).

Cognitive function was assessed by the Symbol Digit Modalities Test (SDMT) and Paced Serial Addition Test 3’ (PASAT). Neuropsychological evaluation was conducted within 24 h prior to the fMRI scanning. The participants were assessed during the same time period of the day, between 10 and 12 am, to eliminate significant circadian variations. Fatigue was assessed by the MFIS. All participants were evaluated by the Beck Depression Inventory (BDI) prior to inclusion. BDI version II, consisting of 21 questions, has a total score that varies from 0 to 63, where 0–10 is considered normal, 11–16 = mild mood disturbances, 17–20 = borderline clinical depression, 21–30 = moderate depression, 31–40 = severe depression, and over 40 = extreme depression. The MFIS consists of 21 questions, including three aspects of fatigue—physical, cognitive, and psychosocial. Total score ranges from 0 to 84, where 38 and over is considered MS-related fatigue syndrome. (9)

A cutoff point of a total score of 55 on the SDMT was used to divide the patients into two groups: cognitively impaired (CI), SDMT score equal to or below 55 points, and cognitively preserved (CP), SMDT score above 55 points, based on the predictive value of the SDMT score proven by Parmenter et al (10).

Participants gave written informed consent prior to any study procedures, and the study protocol was approved by the ethics committee of Medical University of Plovdiv.

### fMRI Acquisition

The scanning of the participants was executed on a 3Т MRI system (GE Discovery 750w) with a protocol including a structural scan (SagT1 3D BRAVO, slice thickness 1 mm, matrix 256 × 256, flip angle 12°) and a functional scan [2D echo planar imaging (EPI), slice thickness 3 mm, matrix 96 × 96, TR (relaxation time) 3,000 ms, TE (echo time) 30, flip angle 90°]. Before each functional scan, five dummy time series were acquired.

The PVSAT (Paced Visual Serial Addition Test) paradigm was applied as a cognitive task during fMRI (11). The PVSAT paradigm consists of two “on” conditions and one “off” condition and a total duration of 11 min 51 s during the fMRI scanning. All “on” blocks are composed of 21 random numbers presented for 3 s each. Before each “on” block, one of the two cues was presented, either “add” or “repeat,” for 3 s. During the “add” condition, the participants were instructed to add each projected number with the previous. During the “repeat” condition, the participants were instructed to silently repeat once each presented number. There were four blocks of each type, alternating between add and repeat followed always by a 21 s “off” block representing a centrally located fixation cross, during which the participants were instructed to look at the cross without thinking of anything in particular (12).

### fMRI Data Analysis

All functional data were analyzed with statistical parametric mapping (SPM12) software running on MATLAB R2017a for Windows. The preprocessing included the following steps: 1) realignment of the functional data in order to correct for head motion; 2) co-registration was conducted between the high-resolution structural image and the functional scans; 3) estimation of spatial registration parameters based on the anatomical image was performed. Consequently, transformation of all co-registered functional data was standardized to MNI (Montreal Neurological Institute) space; those steps were followed by spatial smoothing with a 6 mm full-width-at-half-maximum Gaussian kernel. First-level analysis was then specified, parameters estimated, and t-contrasts defined for all active conditions together and separately vs. the passive condition. The following five contrasts were obtained for each subject: (add+repeat>off), (add>off), (repeat>off), (add>repeat), and (repeat>add). The (add>off) contrast was considered as clinically informative and was used for assessment.

The resulting contrast maps were then used in a second-level random effects analysis to look for the between-group differences, CP vs. CI. The aim was to compare BOLD activations during (add>off) in both groups. The level of significance was set at p < 0.05, FWE (family-wise error) corrected. Regression analysis was used to assess positive and negative correlations between MFIS score and BOLD activations during the cognitive task.

### Statistical Data Analysis

Demographic and clinical characteristics of the subjects were analyzed with SPSS 19.0 for Windows. Normality of distribution was assessed by means of the Kolmogorov–Smirnov test. Between-group analysis of normally distributed data was done by independent sample t-test.

## Results

Demographic, clinical, and cognitive data for all participants is presented in **Table 1**. Comparative statistics was performed between the two groups.

**Table 1:** Demographic, clinical and cognitive data for all participants.

Characteristics	CI (n=30)	CP (n=12)	P
**Age (mean ± SD)**	40.70 ± 7.7	36.92 ± 7.4	0.155
**Education (mean ± SD)**	13.20 ± 2.4	14.42 ± 2.7	0.157
**Disease duration (mean ± SD)**	10.13 ± 4.8	8.42 ± 5.3	0.313
**EDSS (mean ± SD)**	2.200 ± .65	1.625 ± .74	0.017
**SDMT (mean ± SD)**	42.40 ± 9.18	57.83 ±2.56	0.000
**PASAT (mean ± SD)**	34.07 ± 13.72	46.42 ± 11.45	0.009
**MFIS (mean ± SD)**	12.43 ± 12.1	12.17 ± 15.91	0.953
**BDI (mean ± SD)**	4.30 ± 4.94	2.67 ± 4.29	0.322

CI, cognitively impaired; CP, cognitively preserved; EDSS, Expanded Disability Status Scale; SDMT, Symbol Digit Modalities Test; PASAT, Paced Serial Addition Test; MFIS, Modified Fatigue Impact Scale; BDI, Beck Depression Inventory.

**Figure 1:**
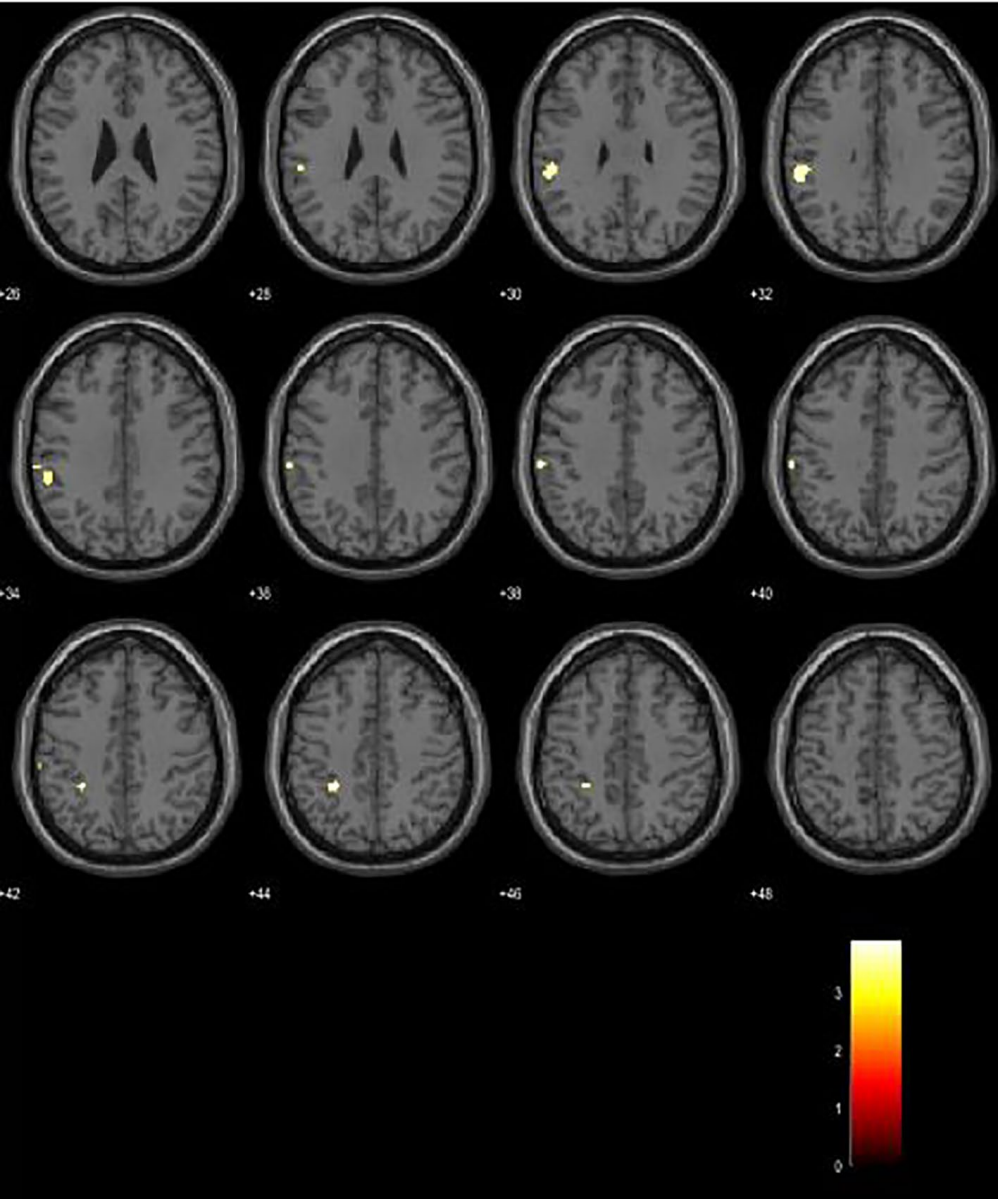
Between-group analysis revealed increased BOLD signal in CP patients.

Between-group analysis revealed increased activations in left Brodmann area (BA) 40 in CP patients, with a significance level of p < 0.001. Analysis yielded several clusters located in the left supramarginal gyrus (BA 40) of a cluster size of 63 voxels, MNI coordinates −56 −36 32, p-value 0.563; a cluster with 24 voxels, MNI coordinates −28 −40 44, p-value 0.848; and a cluster of 22 voxels, MNI coordinates −62 −26 40, with a p-value of 0.863 (**Figure 1**).

**Figure 2:**
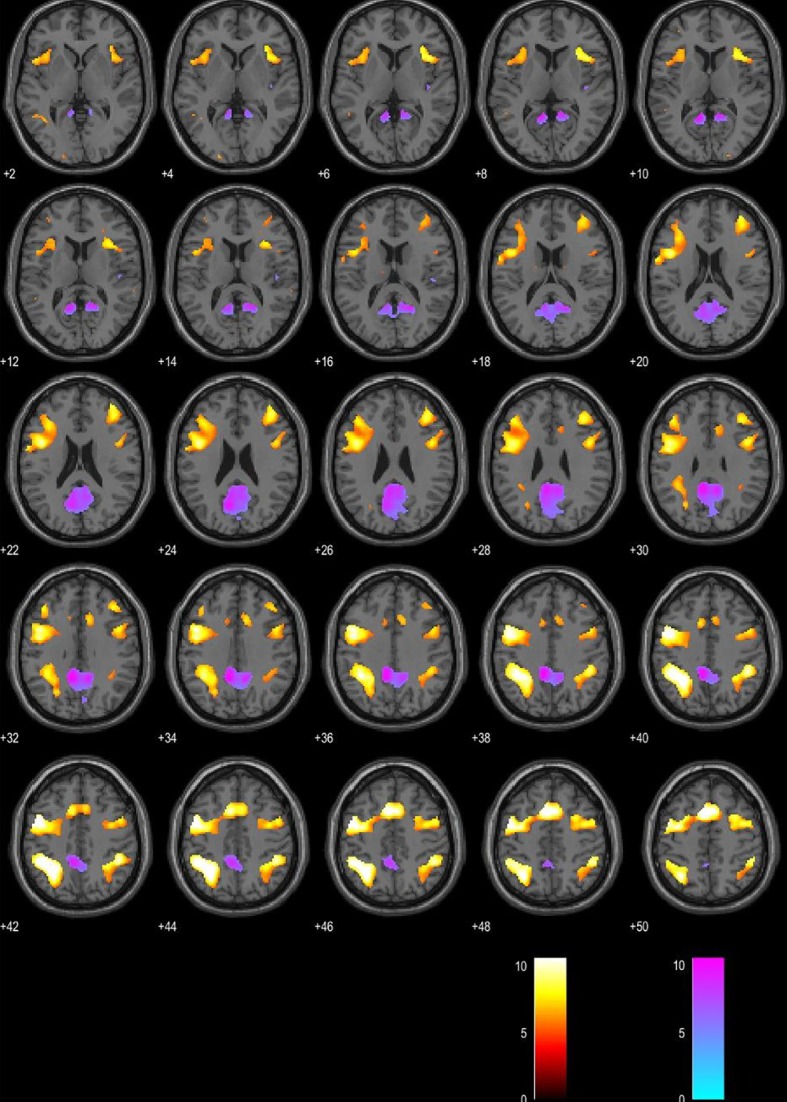
Blood oxygen level–dependent (BOLD) signal associated with higher Modified Fatigue Impact Scale (MFIS) score is presented in the red color map. BOLD signal associated with lower MFIS score is presented in the blue color map. Presented in the axial plane.

**Figure 3:**
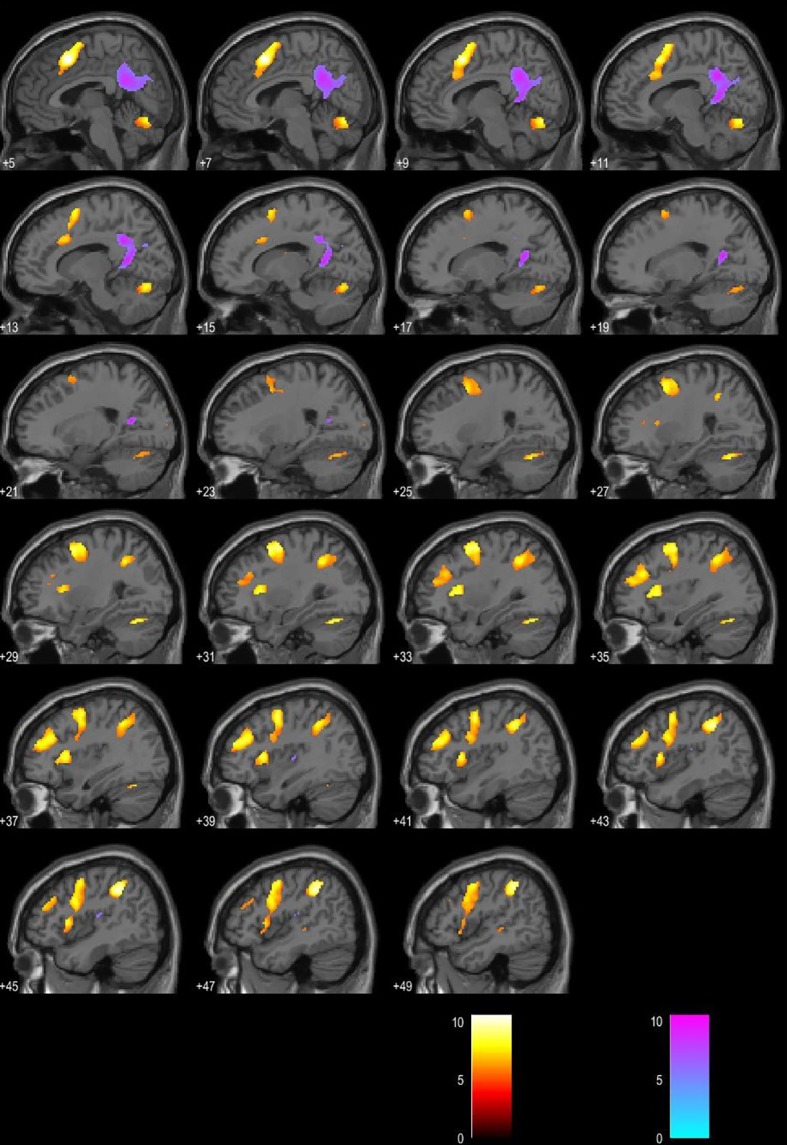
Blood oxygen level–dependent (BOLD) signal associated with higher Modified Fatigue Impact Scale (MFIS) score is presented in the red color map. BOLD signal associated with lower MFIS score is presented in the blue color map. Presented in the sagittal plane.

Regression analysis yielded increased activations in left BA 40, right BA 40 (supramarginal gyrus), and left BA 6 (premotor cortex) in patients with a higher score on MFIS. Stronger BOLD activation in left BA 31 (posterior cingulate gyrus) was associated with a lower score on MFIS. Significance level was set to p < 0.05, FWE corrected. (**Figures 2** and **3**).

## Discussion

The neuropsychological assessment of our subjects confirmed the predictive value of SDMT for cognitive dysfunction as established by Parmenter et al. This is evidenced by the significant difference in performance on the PASAT in both groups (10). Although the use of only one neuropsychological test to categorize our patients may be a limitation, we chose the statistically validated threshold introduced by Parmenter et al. since the SDMT is currently not corrected for age and education within the Bulgarian population. In recent articles, research in MS clearly supports the reliability and validity of the SMDT, and based on current evidence, the test is included as an indispensable method in the recommendations for cognitive screening and management in MS care by the Consortium of Multiple Sclerosis Centers (13, 14).

Interestingly, our study showed no statistical difference in regard to educational background between CP and CI patients. Considering the well-established theory of cognitive reserve, this may not be that unparalleled. According to Sumowski and Leavitt, the reserve against disease-related cognitive impairment consists of both genetic/heritable and environmental factors. Maximal lifetime brain growth (MLBG) is considered a main heritable factor (15). The protective effect of a larger MLBG is based on the “brain reserve” concept explained by Satz, where cognitive decline emerges when brain volume falls below a critical threshold; thus, people with larger MLBG can better withstand disease burden associated with loss of brain volume/brain atrophy without cognitive decline (16). The environmental factor at play is intellectual enrichment, which is largely a product of life experience and is not always linked to education. Educational attainment is often impacted by factors outside our control such as socioeconomic and parental educational status. Research conducted by Sumowski, Rocca, et al. in MS patients concluded that greater early life cognitive leisure protects MS patients from cognitive decline independently of MLBG and education (17).

Contrary to what we initially expected, our results showed no significant difference between CP and CI patients in the mean MFIS score. The mean score on the MFIS in patients was relatively low and did not reach the “clinically significant” cutoff point of 38, adopted by Flachenecker et al (9). However, the regression analysis revealed a notable inconformity in BOLD signal in relationship to the MFIS score. Activations in left and right supramarginal gyrus (BA 40) and left premotor cortex (BA 6) were associated with a higher score on the MFIS questionnaire; on the other hand, the left posterior cingulate gyrus (BA 31) was associated with a lower MFIS score. According to existing data resulting from studies investigating the correlation between fatigue and fMRI, the MFIS scale has not been used so widely in similar research. Most published studies evaluate fatigue with the FSS, self-reported cognitive fatigue by a VAS scale, or physical fatigue after induction with a motor task (18). This differentiation between fatigue domains is pragmatic considering some of the recommendations made by researchers in this field (4, 7). That may be one of the disadvantages of our study. On the other hand, the BOLD clusters revealed in our research are in line with some previous studies. DeLuca et al. investigated neural correlates of cognitive fatigue using fMRI in MS patients. Participants performed a modified version of the SDMT during fMRI acquisition, and cognitive fatigue was defined operationally as an increase in BOLD response across time. The authors hypothesized that patients would show a greater increase in cerebral activity on the cognitive task across time than healthy controls. Among areas with fatigue interaction were BA 40 and BA 19 (3). More recent research by Genova et al. used fMRI to examine where in the brain BOLD activity covaried with “state” fatigue assessed during a task designed to induce fatigue while in the scanner. The authors implemented a subtler approach to the definition of fatigue, where state fatigue refers to a temporary condition which can change over time; on the other hand, “trait” fatigue indicates the opposite. The latter was explored by diffusion tensor imaging (DTI) to investigate white matter integrity. During performance of this cognitively fatiguing task, BOLD activations in BA 6, BA 39, and BA 37 were associated with self-reported state fatigue evaluated by VAS in MS patients (19). It is suggested that depression, mood, and anxiety should be included as covariates when investigating MS-related fatigue. Depression affects a significant part of patients with MS during their life; Bakshi et al. found that depression is associated with MS-related fatigue and should be controlled for (4, 20, 21). In this regard, our fatigue evaluation is valid, since all patients were assessed by the BDI, and the mean score for both subgroups was presented within normal limits.

Between-group analysis comparing BOLD activations in CP vs. CI patients during the PVSAT cognitive paradigm revealed increased activity in our CP patient group, located in the left supramarginal gyrus (BA 40). Our results are in agreement with multiple conducted studies investigating cortical recruitment during cognitive tasks in patients with MS. Since the introduction of functional MRI as a method, it has been extensively applied in neuroscience to illuminate how cortical activation is altered after brain tissue injury (22). Staffen et al. conducted an earlier fMRI research in patients with RRMS with PVSAT paradigm. Compared to healthy controls, the patient group revealed additional cortical recruitment in left BA 6, 8, and 9 and right BA 39 (23). Mainero et al. investigated functional brain activity in patients with RRMS and controls during PASAT and a recall task. The authors observed that fMRI activity was greater in patients with better cognitive function than those with worse cognitive performance and interpreted the data as evidence for compensatory brain reorganization (24). The indication that an increase of cortical activity is demonstrated in patients with preserved cognitive functions, and on the contrary, a decrease in cognitively impaired patients, is well established by several studies (25–27). This reorganization in brain activity is often referred to as proof of brain plasticity; consequently, the question arises whether this mechanism is adaptive or maladaptive. Therefore, researchers compel for longitudinal task-based and resting state fMRI studies with structural MRI data as covariates in order to understand this complicated MS-related cognitive dysfunction (28–31). In light of this, a major disadvantage of our study is the lack of structural MRI data for lesion load and brain atrophy in our subjects. Our study demonstrates that activation of BA 40 both represents a compensatory recruitment in CP patients and is associated with a higher MFIS score. We interpret this overlap as a possible mechanism for diminishing fatigue impact on cognitive functioning in CP patients. Further studies in this direction are necessary in order to understand how preserved cognitive functioning is affected by mood disturbances and fatigue.

Considering the vast concomitant symptoms in MS such as fatigue, anxiety, mood disturbances, and depression, we cannot deny their interdisciplinary nature. Despite the accumulation of data in that direction, these issues are still partly neglected in daily patient management and MS research. Feinstein et al. imply that psychiatrists and neuropsychologists should therefore play a much more prominent role in daily patient management (32). From a scientific point of view, translational neuroscience and its development is essentially a bridge between disciplines in medicine. As observed by Stoyanov, fMRI is an indispensable tool in translational methodology, and by original definition, its purpose is to translate knowledge in different neuroscientific aspects (33, 34).

In conclusion, our study confirms the presence of cortical reorganization and additional cortical recruitment in patients with preserved cognitive function. The impact of fatigue on cortical activation during a cognitive task is demonstrated by inconformity of activated areas depending on the MFIS score. Our results suggest that activation in BA 40 may represent a mechanism for diminishing fatigue impact on cognitive functioning in CP patients.

### Limitations

Using only one neuropsychological test for classifying our patients is a real limitation. The BICAMS (Brief International Cognitive Assessment of MS) is, however, not a validated battery for the Bulgarian population, and it has not been translated in the Bulgarian language. Because of this, we had to circumvent this obstacle as best we could, by incorporating SDMT and PASAT, two tests that are well established for the population we investigate. The cited study conducted by Parmenter et al. compares the SDMT score to the Minimal Assessment of Cognitive Function in MS (MACFIMS) results. Patients were considered cognitively impaired when performing one and a half standard deviations below controls on two or more MACFIMS variables, excluding the SDMT. The authors conclude that the Bayesian statistics showed that a total score of 55 or lower accurately categorized 72% of the patients with a sensitivity of 0.82, specificity of 0.60, positive predictive value of 0.71, and negative predictive value of 0.73. In consequence, we chose to rely on this statistically validated threshold of 55 on the SDMT since the test has not yet been validated and corrected for age and education within the Bulgarian population.

## Data Availability Statement

The datasets generated for this study are available on request to the corresponding author.

## Ethics Statement

The studies involving human participants were reviewed and approved by Medical University Plovdiv Ethical Committee. The patients/participants provided their written informed consent to participate in this study.

## Author Contributions

DI contributed substantially to the design of the work and performed all data acquisition, analysis, and interpretation. AT contributed substantially to the design of the work, performed statistical analysis and interpretation, and provided important feedback and revising. SM contributed to the design of the work, and provided technical methodology and revision. KT contributed to the design of the work, functional technical parameters, and data interpretation.

## Funding

The funding of this research is provided by Medical University Plovdiv.

## Conflict of Interest

The authors declare that the research was conducted in the absence of any commercial or financial relationships that could be construed as a potential conflict of interest.

The handling editor declared a shared affiliation, though no other collaborations, with several of the authors DI, AT, SM, KT at time of review.
